# Enhancing the shelf life of natural scale inhibitors using bio preservatives

**DOI:** 10.1038/s41598-025-90831-5

**Published:** 2025-03-08

**Authors:** E. Khamis, D. E. Abd-El-Khalek, Manal Fawzy, Habiba M. Essam, A. M. Abdel-Gaber, J. M. Anwar

**Affiliations:** 1 Chemistry Department, Faculty of Science, Alexandria University, Alexandria, 21321 Egypt; 2 Science & Innovation Center of Excellence, SICE, Egyptian Russian University, Badr, Egypt; 3 National Institute of Oceanography and Fisheries (NIOF), Cairo, Egypt; 4 Environmental Sciences Department, Faculty of Science, Alexandria University, Alexandria, 21511 Egypt; 5 Green Technology Group, Faculty of Science, Alexandria University, Alexandria, 21511 Egypt; 6 Water Company, Holding Company of Water & Wastewater, P.O. Box: 21511, Alexandria, Egypt

**Keywords:** Green scale inhibitor, Shelf life, Natural extract, EIS, *Salvia Rosmarinus*, Ecology, Environmental sciences, Chemistry

## Abstract

One of the key challenges in using natural extracts for water treatment is their biodegradability and susceptibility to microbial spoilage, which can limit storage and long-term effectiveness. This study investigates the scale inhibition capabilities of an aqueous extract of *Salvia rosmarinus***sp** through electrochemical measurements, conductivity tests, and morphological examination. Additionally, two natural substances, Rhamnolipid and Chitosan, were evaluated as bio-preservatives to prevent mold growth and enhance the shelf life of the rosemary extract. The reasons for selecting these specific bio-preservatives include their known antimicrobial properties, antioxidant effects, environmental benefits, and suitability for the intended application. For 24 weeks, we conducted a microbial examination and assessed the anti-scaling performance of the extract in combination with the bio-preservatives. The results demonstrate that rosemary extract significantly inhibits CaCO_3_ scale precipitation, attributed to the presence of carboxylate and hydroxyl groups which effectively chelate cations and disturb the normal crystal growth of the scales. Additionally, the rosemary extract-chitosan mixture exhibits superior antimicrobial and anti-scaling performance compared to the rosemary extract–rhamnolipids combination over six months. It can be concluded that a 1:2 ratio of chitosan to rosemary extract provides an effective eco-friendly scale inhibitor and reduces the growth of pathogenic bacteria and fungi with an extended shelf life. In this context, biosurfactants and polysaccharides present beneficial properties that offer sustainable and biological alternatives to conventional chemical biocides.

**Supplementary Information:**

The online version contains supplementary material available at 10.1038/s41598-025-90831-5.

## Introduction

Scaling refers to the accumulation of sparingly soluble inorganic salts in water-supply systems and industrial applications. This buildup can lead to complete or partial blockages in equipment and pipes, resulting in significant issues such as corrosion, energy loss, economic setbacks, and potential accidents that compromise production safety^1–4^. A widely used approach to mitigate scale deposition is the application of antiscalants. Scale inhibitors (SIs) function by disrupting the formation of calcite film, effectively preventing the precipitation, deposition, and growth of scales on solid surfaces^5^.

Phosphorus-containing antiscalant compounds are among the most effective chemicals available commercially. However, these phosphorus-based scale inhibitors have significant drawbacks, including prolonged biodegradation in water^6^. The presence of inorganic or organic phosphonate in these scale inhibitors raises phosphorus levels in water, contributing to severe eutrophication^7–9^. Additionally; many phosphorous compounds are toxic and costly.

Increasing environmental concerns and stricter discharge regulations have driven the development of eco-friendly antiscalants that are easily biodegradable and have minimal environmental impact. Recent intensive efforts have focused on creating sustainable alternatives to organophosphates and nonbiodegradable polyacrylates^10–12^. Among these novel inhibitors, polymaleates (PMA), polyaspartates (PASP), and polyepoxysuccinates (PESA), along with their various derivatives, including copolymers with polyacrylic acid (PA), show great promise.

Recently, Plant extracts have emerged as an interesting alterntransfer resistances of the steel electrode ative source of scale inhibitors, offering an environmentally friendly option that is easy to prepare^13^, Their biodegradability, affordability, renewable nature, and ease of application make them particularly appealing. Utilizing plant extracts to inhibit scale formation is considered a sustainable and eco-friendly approach, as many phytochemicals are water-soluble metabolites, including organic acids, quinones, phenolic compounds, flavonoids, alkaloids, catechins, terpenoids, and co-enzymes. Furthermore, these extracts also contain amino acids, plant-derived proteins, polysaccharides, and vitamins, all of which have no adverse effects on living organisms or the environment^14^.

Many plant extracts have demonstrated effective anti-scaling properties, including the hull and leaves of *Punica granatum* extract^15,16^, palm leaves extract^16,17^, *Bistorta officinalis* extract^18^, clove extract^19^ and brown seaweed extract^20^.

However, the biodegradability of plant extracts poses challenges for their storage and long-term use. Microbial decomposition of these extracts can be mitigated by incorporating biocides and other stabilizing agents. Recent studies have explored the blending of plant extracts with biocides to create effective scale inhibitors for extended use^21^. For example, Reno and Endaryanto^22^ investigated the addition of 1 gm of benzoic acid and 2 gm citric acid as biocides to powdered Gambier extracts. Similarly, Viloria et al.^23^ developed a scale inhibitor blend using Aleo Vera gel and methanol.

This study aims to extend the shelf life of scale inhibitors derived from natural extracts using bio-preservatives. It focuses on evaluating the effectiveness of the aqueous extract of rosemary (*Salvia rosmarinus)* as an antiscalant for the CaCO_3_ scale through electrochemical techniques and beaker tests. The safety of rosemary is previously reported due to the presence of numerous active ingredients identified in rosemary extracts that exhibit physiological properties such as antiviral, antibacterial, antioxidant, and anticancer effects^24^. Additionally, the research investigates the impact of incorporating Rhamnolipids and Chitosan as two bio-preservative materials on controlling pathogenic bacterial and mold formation and prolonging the shelf life of rosemary extract. Rhamnolipids, naturally derived biosurfactants, are widely used in the food industry to enhance product quality and prolong the shelf life of products^25^ due to their various functional attributes, including stabilization, antibacterial activity, antioxidant activity, and emulsifying ability. Similarly, Chitosan exhibits significant self-antioxidant^26^ and self-antibacterial properties^27,28^, making it a promising candidate for preventing contamination and microbial spoilage in the food industry^29^.

## Results and discussion

### FT-IR examination of aqueous extract of *Salvia Rosmarinus*

**Fig. 1:**
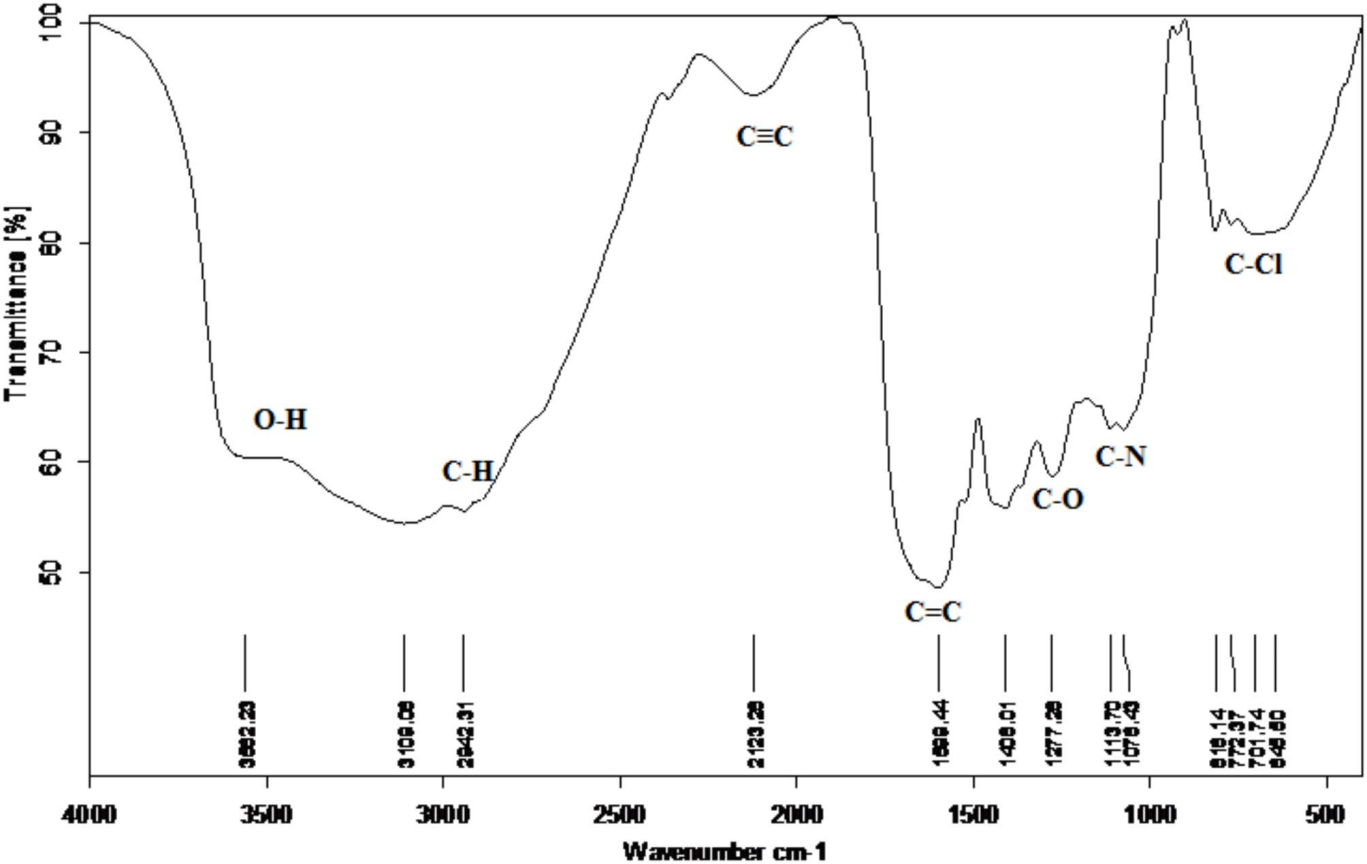
FTIR spectra of Rosemary extract in the region of 500 to 4000 cm^− 1^.

To investigate the main functional groups existing in rosemary extract and the possible mechanism of its anti-scaling performance, FT-IR examination was done. Figure 1 represents the bands found in the examined range. As seen, eight fingerprints at the following absorption zones: 3500 − 3300, 2900 − 2700, 2150 − 2100, 1650 − 1600, 1500 − 1400, 1320–1000 cm-1, and 1000–1100 cm-1. 700–610 cm-1. The broad band at 3562.2 is probably associated with O-H stretch and H-bonded of carboxylic acids, phenols and alcohol which is assigned to phenolic hydroxyl group OH stretching vibrations. While the broad band at 2942.3 cm-1 is most likely representing the alkanes’ C–H stretch. The broadband at 2123.2 cm^− 1^ is related to C–C stretch of alkenes. The absorption bands at 1599.4 cm ^-1^ was measured which associated with the stretching vibration of aromatic rings at the C = C position^30–32^ as well as the vibration of amines’ N–H bond, amides’ C = O bond, and carboxylic groups^33,34^ The stretching vibration at band 1408 cm^− 1^ is associated with the stretching vibration of aromatic rings at the C = C position. The intensity increase of the absorption band between 1277,1113 cm^− 1^, as seen in the spectra of methylated and acetylated lignin, allows us to relate this band to asymmetric stretching vibrations of the C-O-C linkages in phenolic ethers (C-O stretch) and esters or to phenolic hydroxyls^35^, while the broad band at peak 1076.4 cm^− 1^, which is most likely related to the C–N stretching in aliphatic amines. Broad band of peak 816.1 cm^− 1^, 772.6 cm-1 and 645.5 cm-1 indicated the presence of. C-Cl stretch and C-Br and the association of alkyl halide^36^. According to these results, the main bands detected in the rosemary extract are attributed to the presence of hydroxyl groups (O-H), carboxylic groups (COOH), and alcoxy groups (C-O), which are related to the different flavonoids and phenolic compounds that are present. The combination of these negatively charged groups and cations (Ca^2+^), which inhibit the normal formation of CaCO_3_ crystals, may be responsible for the antiscalant effect of rosemary extract.

### Performance of *Salvia Rosmarinus* extract as calcium carbonate scale inhibitor

#### Chronoamperometry measurements

**Fig. 2:**
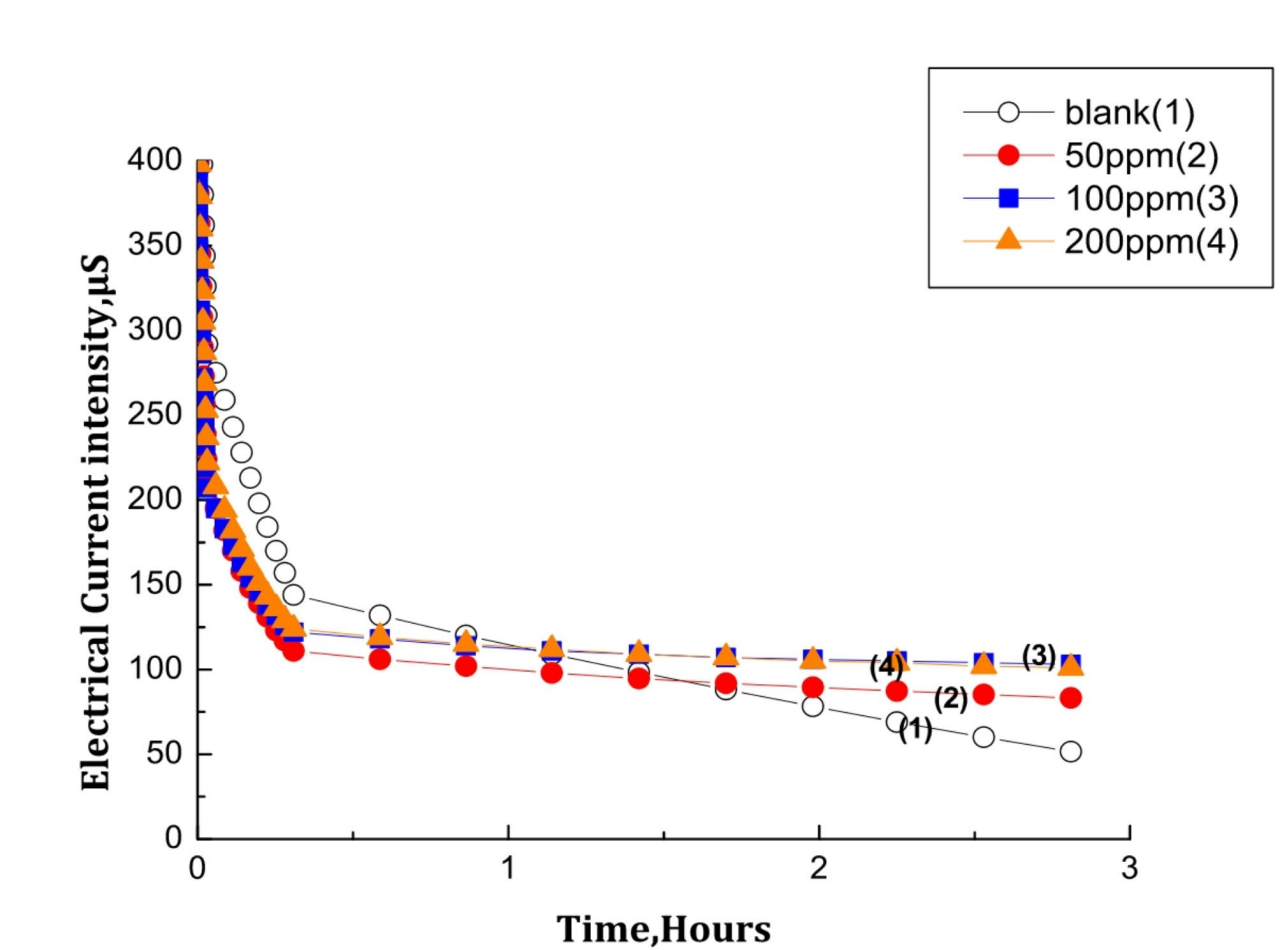
Chronoamperometry curves for polarized steel electrode in scaling solution without and with varying concentrations of rosemary extract at pH = 7.37 and 40^o^ C.

The chronoamperometry curves resulting in cathodic polarization of steel electrode in brine solution in the absence and presence of different rosemary extract concentrations are presented in Fig. 2. The sharp decrease in the current density in the absence of the extract indicates that the nuclei of CaCO_3_ were growing and occupied the steel surface. On the other hand, the current density decreases slowly in the presence of the extract. Moreover, the residual current density increases with increasing the rosemary extract concentrations indicating that fewer CaCO_3_ crystals were precipitated on the steel surface and proved its effectiveness as an anti-scalant.

#### Impedance measurements

**Fig. 3:**
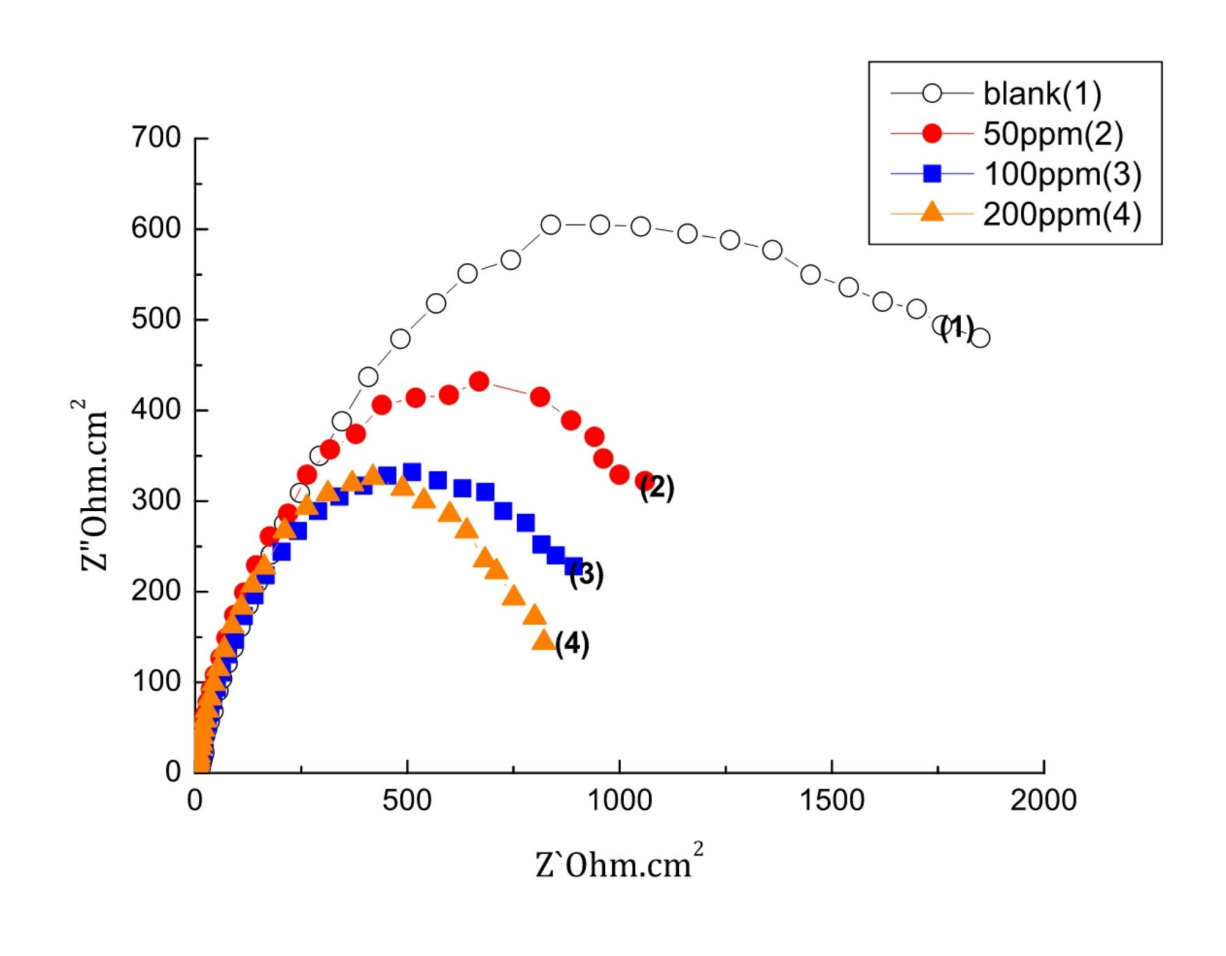
Electrochemical Impedance Spectra of steel in scaling solution without and with different Rosemary concentrations at pH = 7.37 and 40^o^ C.

Impedance spectra for the steel following CaCO_3_ precipitation using cathodic polarization in scaling solution both in the absence and in the presence of different rosemary^,^ s concentrations are shown in Fig. 3. The Nyquist diagrams display a characteristic of depressed semi-circles. As can be observed, the presence of the extract causes a decline in the size of the deformed semicircles because it reduces the charge transfer resistance, which lowers the scale’s insulation layer^37^. Table 1 represents the results derived from the impedance spectrum’s computer fitting to the equivalent circuit shown in Supplementary data (Fig. S1). This equivalent circuit was employed to analyze the experimental data from the impedance plots. This equivalent circuit, earlier proposed by Beaunier et al.^38^, was employed to analyze the experimental data from the impedance plots. According to the results, the charge transfer resistance R_ct_, which is inversely proportional to the quantity of scale formed, decreased as rosemary concentrations increased. This behavior reflects this substance’s ability to inhibit scale formation^39^.

**Table 1:** The computer-fitted results of the impedance spectrum of the steel electrode, which was polarized cathodically for three hours in a CaCl_2_ brine solution with varying concentrations of Rosemary extract after 3 h at 40^o^ C.

Conc., ppm	*R*_s_ ohm.cm^2^	Q µF	*n*	*R*_ct_ Ohm.cm^2^	% of Inhibition
0	9.148 ± 0.074	149.9 ± 0.836	0.675	2207 ± 25.1	--------
50	6.29 ± 0.042	193.2 ± 1.125	0.808	1202 ± 12.08	45.5%
100	6.83 ± 0.041	250 ± 1.52	0.834	841 ± 9.25	61.9%
200	5.918 ± 0.039	303.9 ± 1.97	0.801	924 ± 10.02	58.1%

The following equation can be utilized to calculate the percentage of scale inhibition^39^.

1 $$\% {\text{ }}Scale{\text{ }}Inhibition{\text{ }} = {\text{ }}\left[ {\left( {Rct} \right)_{o} - {\text{ }}\left( {Rct} \right)_{i} } \right]{\text{ }}\left( {Rct} \right)_{o} \times {\text{ }}100$$

where (Rct)o and (Rct)i are the charge transfer resistances of the steel electrode following three hours of cathodic polarization at − 0.95 V (vs. SCE) in the scale environment without and with the tested inhibitor, respectively. Figure 3 illustrates that the maximal CaCO_3_ scale inhibition is about 62% in the presence of 100ppm of rosemary extract. This suggests that the inhibitor caused the carbonate crystals to become distorted and prevented them from adhering to the electrode surface. The good anti-scaling performance of rosemary extract can be attributed to its high functional group content, as seen in Fig. 1.

#### Conductivity measurements

**Fig. 4:**
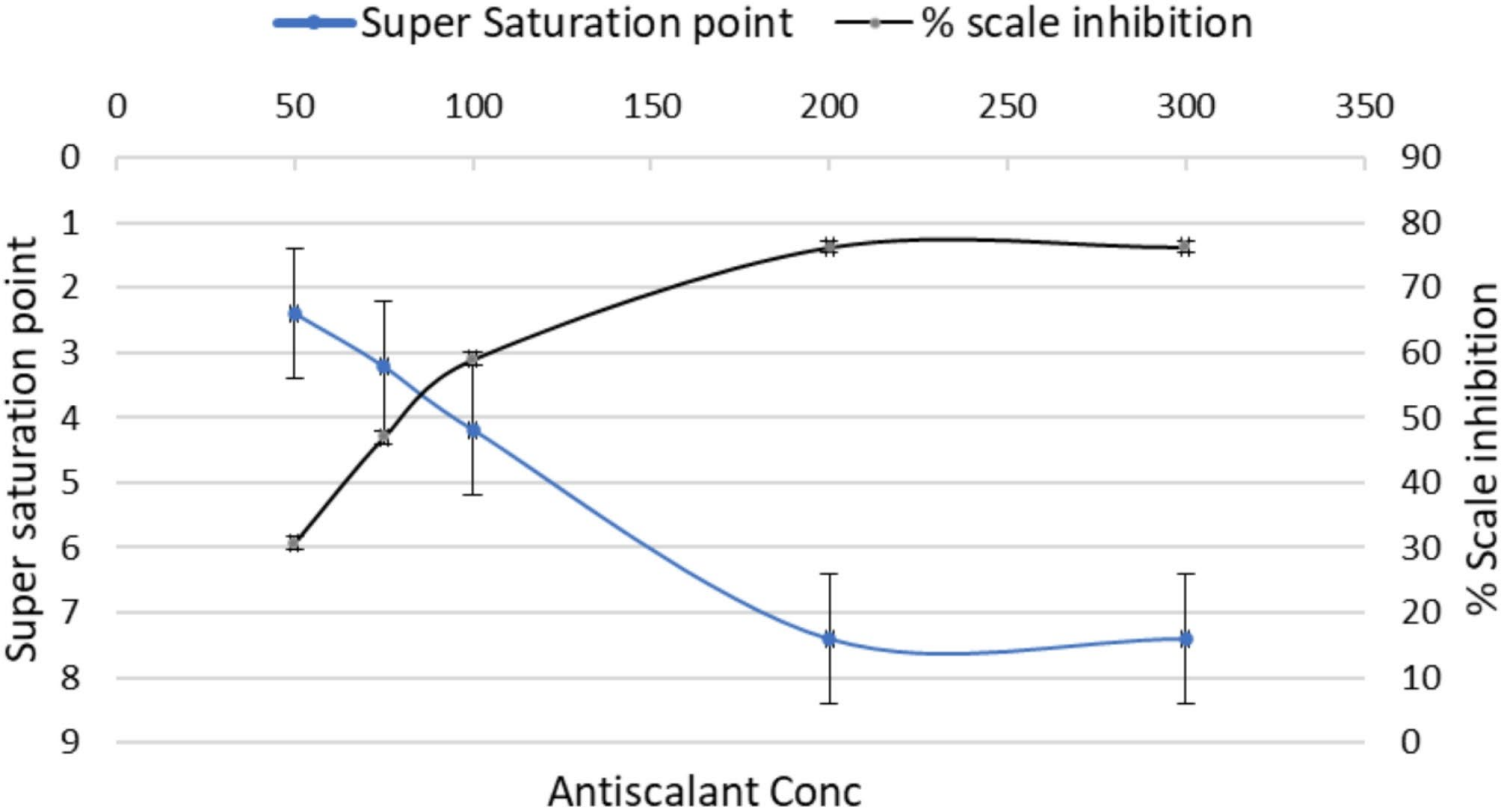
Variation of supersaturation point and % scale inhibition of Calcium Carbonate at different Rosemary extract concentrations at 25 °C and pH = 6.03.

In this test, sodium carbonate was added to calcium chloride solution to precipitate calcium carbonate while observing the electrical conductivity (see Supplementary Data Fig. S2). The conductivity test can be considered as a screening procedure used to evaluate the ability of an inhibitor to impede or prevent the development of critical nuclei in supersaturated solution. Nucleation is recognized as the initial stage of precipitation. The important point in homogeneous nucleation is the supersaturation point or S. The homogenous nucleation rate abruptly increases when the ion product reaches this particular point, causing scaling and precipitation^37^. Figure 4 shows the dependence of the calcium carbonate scale’s supersaturation point at various Rosemary extract concentrations. It can also be seen that the addition of Rosemary extract delays the point of supersaturation, which in turn prevents calcium carbonate precipitation. This is because the extract can suppress or delay the development of the crucial nuclei in the supersaturated fluid. These results demonstrate that adding rosemary extract to the solution significantly lengthens the induction time compared to the same experimental conditions without any additives. Figure 4 also, illustrates the variation of percentage inhibition of calcium carbonate scales calculated from the supersaturation point (maximum of the curve) at different concentrations of extracts using the following Eq. 1 ref^6^:

2 $$\% {\text{ }}Scale{\text{ }}inhibition{\text{ }} = {\text{ }}\left[ {\left( {S_{i} - S_{o} } \right)/S_{i} } \right] \times 100$$

where So and Si are the supersaturation point in the absence and presence of the extract^40^.

As seen in Fig. 4, by increasing the concentration to 200 ppm, the efficiency of extract inhibition increased to 78.3%, but no discernible increase in scale inhibition percent was seen beyond this concentration. This result also agrees with similar outcomes found in previous results^41^. These results reveal that the extract liquefies suspended solids and slows the growth of CaCO_3_ crystals. This indicates that the extract prevents supersaturation, which may be attributed to the adsorption of extract molecules to the active sites of the forming crystals^19,42^.

### Characterization of CaCO3 precipitate

#### Scanning electron microscopy (SEM) of CaCO3 precipitate

**Fig. 5:**
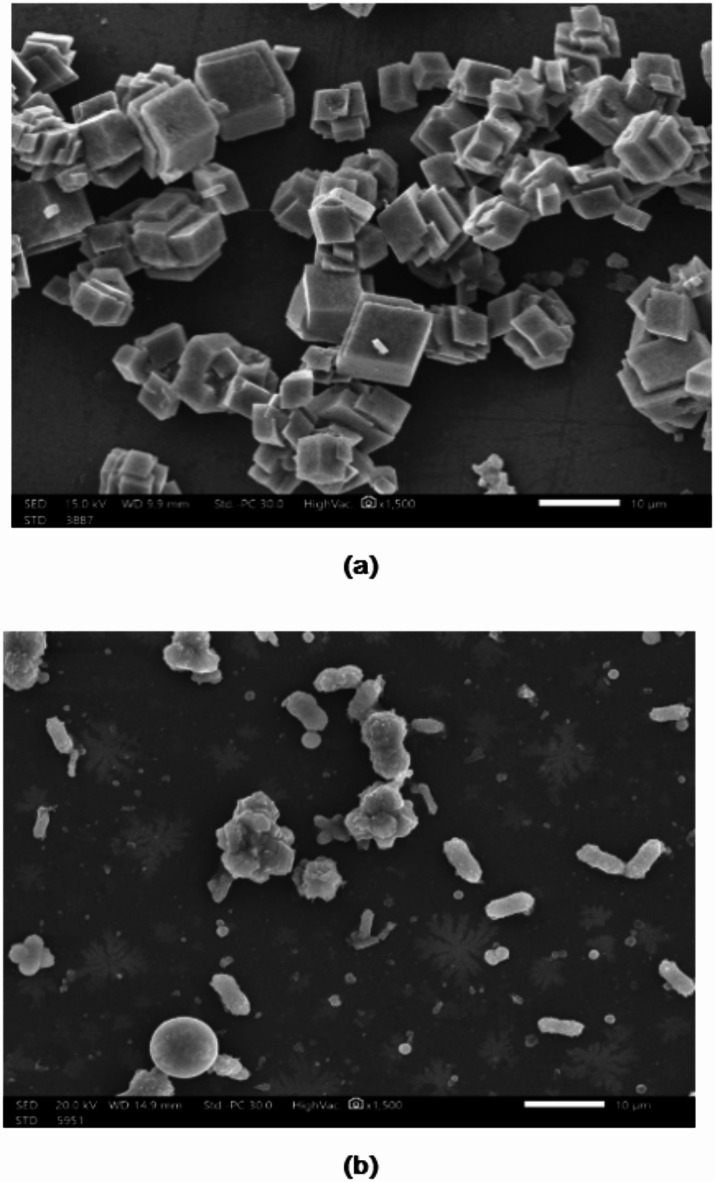
SEM images of CaCO_3_ crystals in the absence (**a**) and presence of Rosemary extract (**b**).

SEM images of CaCO_3_ crystals after precipitation are displayed in Fig. 5. As seen, in the absence of the extract, CaCO_3_ crystals exhibited a standard cubic structure. Conversely, the presence of the extract resulted in the formation of highly irregular and distorted crystals. This can be explained by the many functional groups that are included in the extract, which are adsorbed onto the CaCO_3_ crystal lattice. The particle surface charge density and the repulsive force between the particles increased, thus reducing the growth rate of CaCO_3_ crystals and inhibiting their formation^43^.

#### XRD examination

**Fig. 6:**
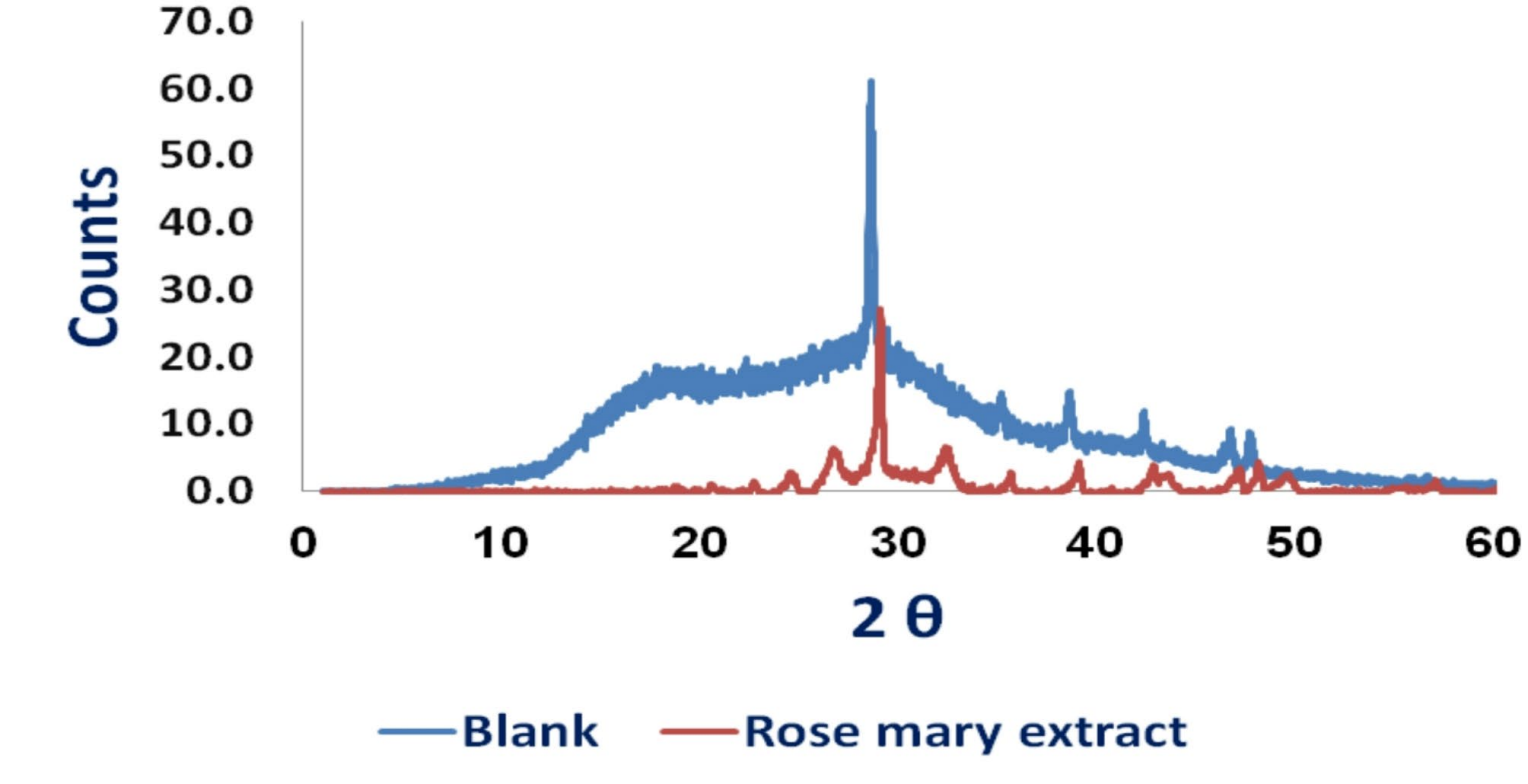
XRD image of the CaCO_3_ crystal formed in the absence and presence of Rosemary extract.

Powder XRD analysis was carried out for further investigation of CaCO_3_ crystallinity. Figure 6 depicts the XRD patterns of CaCO_3_ obtained with and without extract addition. As shown, in the absence of extract, only sharp calcite reflections appeared (peaks at 22.90 (012), 29.28 (104), 31.22 (006), 35.83 (110), 39.30 (113), 42.93 (202), 47.44 (018), and 48.32 (116)), which coincides with the structure of calcite crystal standard feature^44^. The addition of the inhibitor caused the typical peaks to diminish, indicating that the CaCO_3_ particle size and surface shape had changed. As a result, when the inhibitor was added, the crystallinity of CaCO_3_ was reduced^37,43^. Therefore, the extract modified CaCO_3_ crystals as displayed in the SEM. Investigation.

#### Investigation of scale inhibition performance of tested bio-preservatives

Two green bio-preservatives were tested to examine their effectiveness in prolonging the shelf life of the rosemary extract as a natural antiscalant and preventing microbial spoilage. These bio-preservatives include Rhamnolipids (lipids) and Chitosan (polysaccharides).

**Fig. 7:**
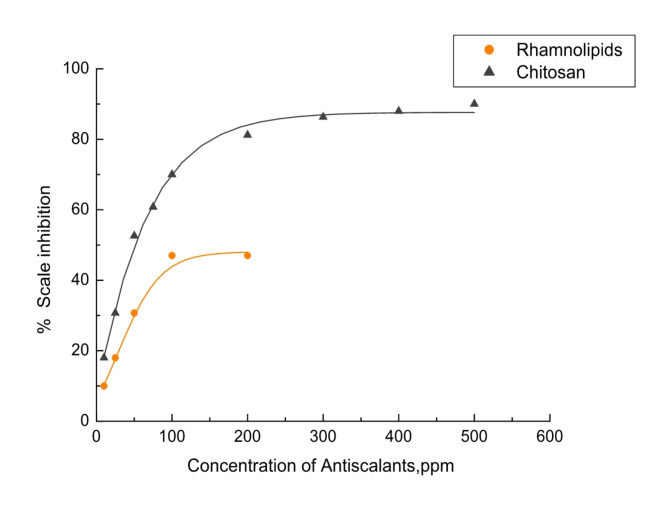
Comparison of calcium carbonate scale percentage inhibition in the presence of Chitosan or Rhamnolipids at 25 °C.

Each of the two proposed green bio-preservatives was individually tested as an inhibitor of CaCO_3_ to assess their effectiveness as a CaCO_3_ antiscalant. This was illustrated in Fig. 7, which compares the percentage of inhibition at various concentrations of the proposed green bio-preservatives under investigation. As seen, Chitosan and Rhamnolipids have a higher antiscalant efficiency of 80 and 47%, respectively.

#### Examination of the rosemary extract-chitosan mixture’s shelf life

The effectiveness of adding the two bio-preservatives in extending the shelf life of rosemary extract was examined. This study selected 200 ppm, which is the optimum concentration of rosemary extract. Two concentration ratios were examined based on the concentration of the bio-preservatives added. The concentration ratios (20:1) and (2:1) correspond to the addition of low (10 ppm) and high (100 ppm) concentrations of the bio-preservative, respectively. The trial was conducted with a 20:1 ratio of Rhamnolipids or Chitosan to reduce the cost of the mixture. All solutions were stored at 4^o^ C.

**Fig. 8:**
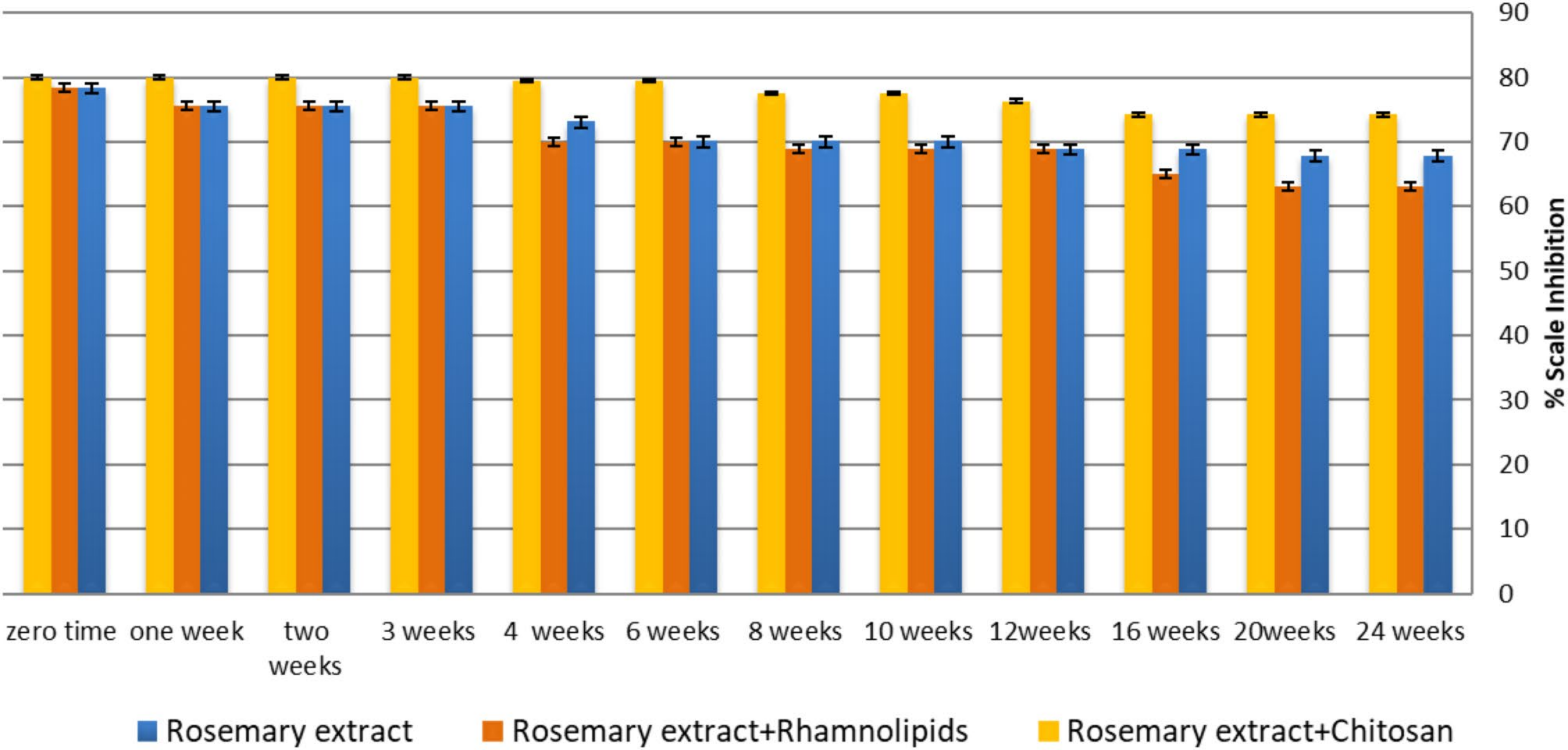
The variation of scale inhibition percentage for rosemary extract in the absence and presence of tested bio preservatives after 6 months in the ratio (20:1) stored at 4^o^ C.

According to Fig. 8 and Table S1(supplementary data), there was a notable decline in the efficiency of rosemary extract after the 6th week, decreasing from 78.3 to 70%, followed by a gradual decrease over time. This decrease in the inhibition performance may be attributed to the rotting of the extract over time. The addition of 10 ppm rhamnolipids exhibited a similar trend to rosemary extract, with an efficiency decrease in the 16th week compared to the efficiency of rosemary extract alone. Conversely, the addition of 10 ppm Chitosan enhanced the performance of rosemary extract as a scale inhibitor, yielding 80% inhibition compared to 78.3% with rosemary alone at zero time. Furthermore, 10 ppm chitosan sustained the efficiency of rosemary after 24 weeks yielding 74.2%, in contrast to the 67.8% efficiency of rosemary extract alone. This superior effect of chitosan could be related to its antioxidant and anti-bacterial activity^26–28^.

**Fig. 9:**
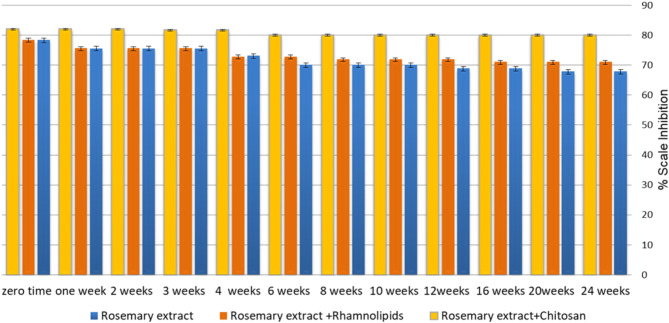
The variation of scale inhibition percentage for rosemary extract in the absence and presence of tested bio preservatives after 6 months in the ratio (2:1) stored at 4^o^ C.

**Fig. 10:**
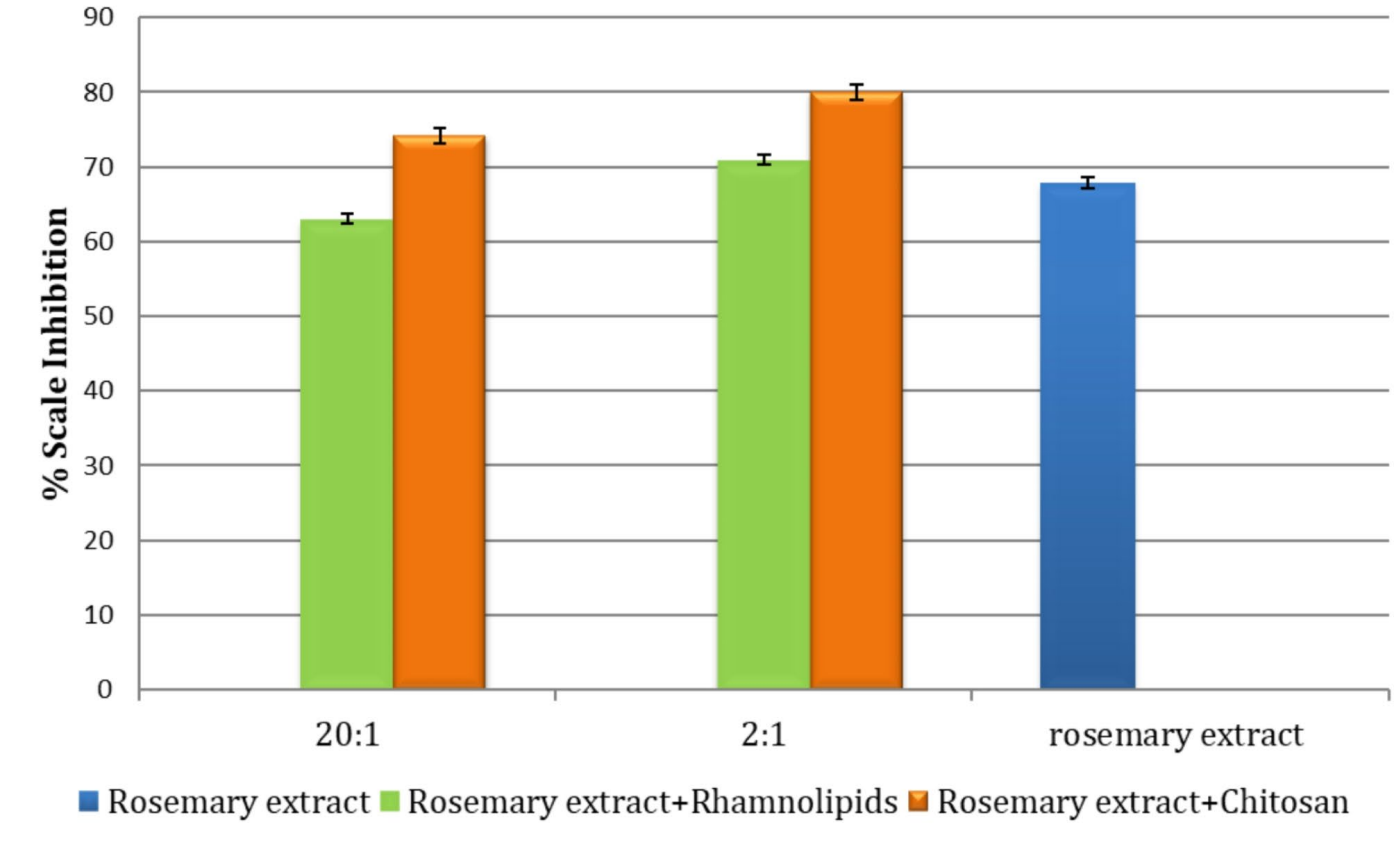
The variation of the scale inhibition percentage of rosemary extract in the presence of different ratios of tested bio preservatives after six months stored at 4^o^ C.

On the other hand, as illustrated in Fig. 9, Table S2(supplementary data), the addition of 100 ppm rhamnolipids maintained the efficiency of the extract until the 16th week, with a decrease in the percent inhibition to 70.9% by the end of the 24th week. On other hand, adding 100 ppm of Chitosan improves the performance of rosemary extract as a scale inhibitor to reach 80% compared to 67.8% of rosemary alone after 24 weeks. Figure 10 displays the variation of the percentage of scale inhibition for rosemary extract in the presence of different ratios of the tested bio-preservatives after 24 weeks. The results showed that the addition of the 2:1 ratio of rosemary to bio-preservatives demonstrated a better efficiency than the 20:1 ratio after 24 weeks. Therefore, the microbiological examination was conducted using the 2:1 ratio for further analysis to test the efficiency of the added bio-preserver to prevent bacterial and mold formation.

Overall, the results indicated that the addition of chitosan to the rosemary extracts, with the composition ratio rosemary extract: chitosan 2:1 (200 ppm: 100 ppm), represents a mixture of highly qualified green scale inhibitors. This mixture inhibited the formation of the CaCO_3_ scale with an efficiency of 82% at zero time and 80% after six months at 4^o^ C.

The statistical comparison of scale inhibition efficiencies obtained from rosemary alone, rosemary-rhamnolipid, and rosemary-chitosan treatments was studied using both one-way ANOVA test “Tukey’s HSD” and t-tests at 95% Confidence Interval (CI) utilizing Minitab program “Version 20.4”, Table2 illustrates the statistical parameters for scale inhibition efficiency of Rosemary extract, Rosemary-Rhamnolipids and Rosemary-Chitosan mixture at confidence level of 95%, while Table 3 provides the analysis of variance (ANOVA) results for scale inhibition of rosemary in the presence of Rhamnolipids and Chitosan. These findings indicate that:

I. Effect of Rhamnolipids on Rosemary’s Inhibitory Activity:

- A comparison between Rosemary alone and Rosemary + 100 ppm Rhamnolipids yielded a t-value of 2.96 and a p-value of 0.098.
- Since p-value > 0.05, the difference is not statistically significant, suggesting that the addition of 100 ppm Rhamnolipids does not significantly enhance the inhibitory effect of Rosemary.

II. Effect of Chitosan on Rosemary’s Inhibitory Activity:

- A comparison between Rosemary alone and Rosemary + 100 ppm Chitosan resulted in a t-value of 20.25 and a p-value of 0.002 (*p* < 0.05).
- This indicates a statistically significant increase in inhibition efficiency suggesting that incorporating Chitosan enhances rosemary’s inhibitory performance, demonstrating its potential as an effective optimizer for scale inhibition.

**Table 2:** Statistical parameters of scale inhibition efficiency of Rosemary extract, Rosemary-Rhamnolipids and Rosemary-Chitosan mixture at confidence level of 95%.

Inhibitor	Mean	StDev	95% CI (low)	95% CI (High)
200 ppm Rosemary (After 24 weeks)	68.350	0.778	66.188	70.512
Rosemary vs. Rosemary + 100 ppm Rhamnolipids (After 24 weeks)	70.450	0.636	68.288	72.612
Rosemary vs. Rosemary + 100 ppm Chitosan (After 24 weeks)	80.200	0.283	78.419	81.981

**Table 3:** Analysis of variance (ANOVA) data for scale inhibition of rosemary extract in the presence of Rhamnolipids and Chitosan.

Comparison	Difference of means	SE of Difference	t-value	*P*-value	Interpretation
Rosemary vs. Rosemary + 100 ppm Rhamnolipids (After 24 weeks)	2.1	0.711	2.96	0.098	No significant difference (*p* > 0.05)
Rosemary vs. Rosemary + 100 ppm Chitosan (After 24 weeks)	11.8	0.585	20.25	0.002	Significant difference (*p* < 0.05)
Rosemary + 100 ppm Rhamnolipids vs. Rosemary + 100 ppm Chitosan(After 24 weeks)	9.75	0.492	19.80	0.011	Significant difference (*p* < 0.05)

A direct comparison between Rosemary + 100 ppm Rhamnolipids and Rosemary + 100 ppm Chitosan yielded a t-value of 19.80 and a p-value of 0.011 (*p* < 0.05). This confirms that Chitosan is significantly more effective than Rhamnolipids in enhancing Rosemary’s inhibitory potential.

While Rhamnolipids shows some potential to improve Rosemary’s scale inhibition efficiency, the effect is not statistically significant at the given concentration. In contrast, Chitosan significantly enhances the inhibition efficiency, making it a more effective additive for optimizing Rosemary’s performance.

**Fig. 11:**
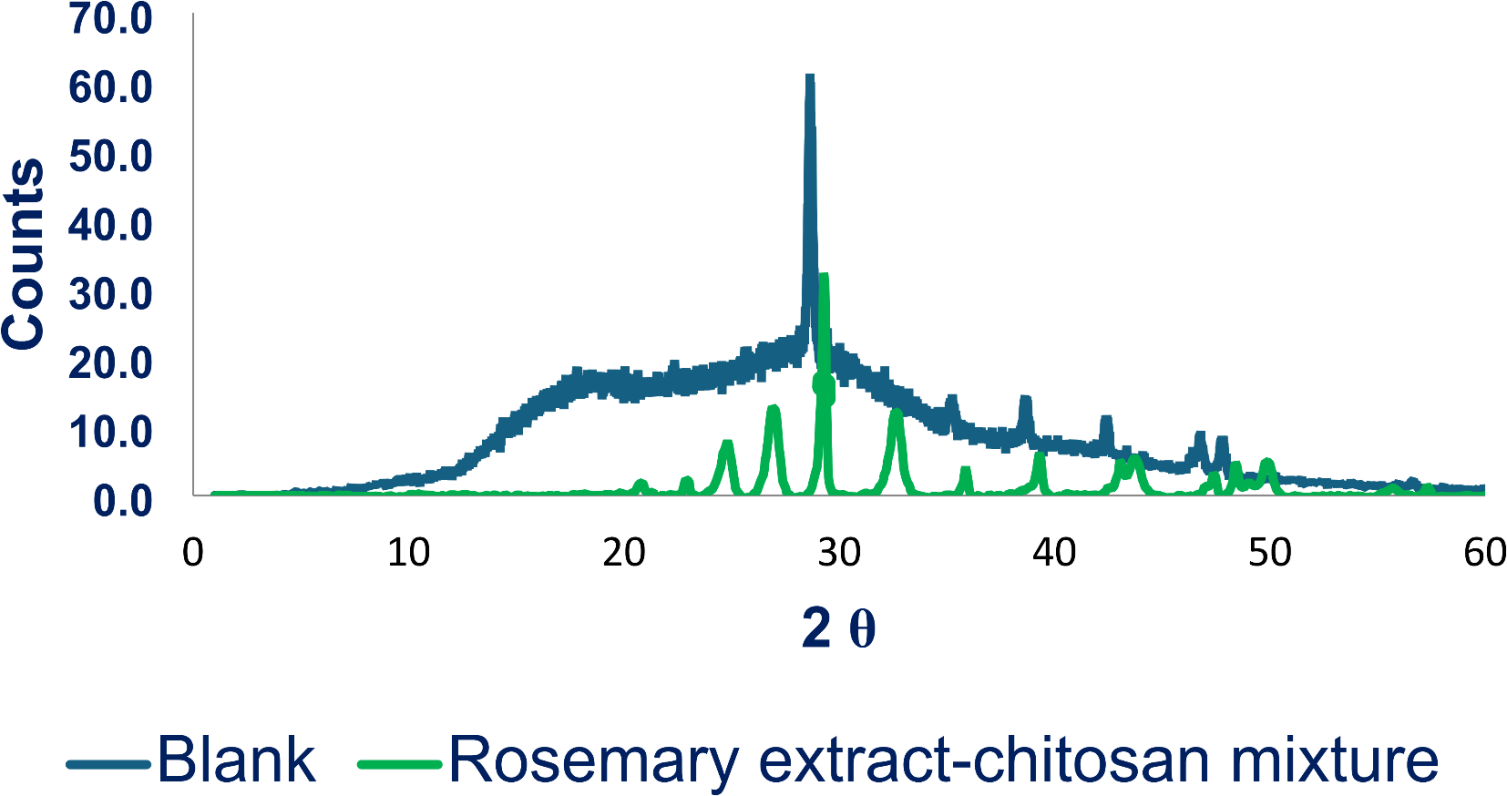
XRD image of the CaCO_3_ crystal formed in the absence and presence of rosemary extract-chitosan mixture.

**Fig. 12:**
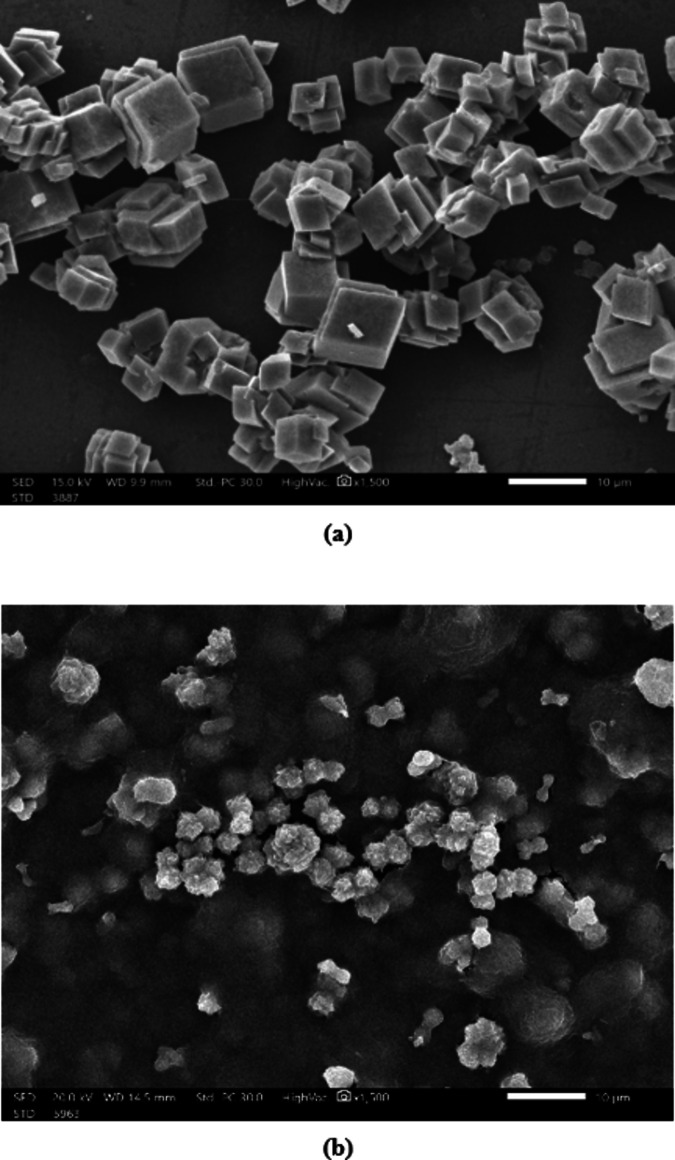
SEM images of CaCO_3_ crystals in the absence (**a**) and presence of rosemary extract-chitosan mixture (**b**).

The X-ray diffraction (XRD) analysis of calcium carbonate (CaCO_3_) samples with and without the presence of the rosemary extract-chitosan mixture is illustrated in Fig. 11. The figure revealed that the characteristic peaks of CaCO_3_ were diminished in the presence of a rosemary extract-chitosan mixture. This suggests that the surface morphology and particle size of CaCO_3_ were altered, leading to a reduction in the overall crystallinity of CaCO_3_ when the mixture was introduced^45^. As a result, the rosemary extract-chitosan mixture modified effectively the CaCO_3_ crystals and impeded their adsorption onto equipment surfaces, as supported by the SEM images depicted in Figs. 12^46,47^.

The comparisons of the scale inhibition between Rosemary extract and some other naturally-based scale inhibitors are listed in Table 4 ref^15–20,23,34,39^. It is believable that a rosemary-chitosan mixture can be considered a promising green-scale inhibitor.

**Table 4:** Comparisons of the scale inhibition between Rosemary extract and some other naturally-based scale inhibitors at zero time.

Scale inhibitor	Scale type	Test method	Dose	Inhibition efficiency (%)	Reference
Rosemary extract- chitosan mixture	CaCO_3_	Electrochemical measurements Conductivity test	(200:100) ppm	82	Recent study
Fig leaf extract	CaCO_3_	Electrochemical measurements	75 ppm	85	(38)
Aloe vera	CaCO_3_	Field tests	15.2 mg/L	Efficient	(23)
Punica granatum hull	CaCO_3_	Electrochemical measurements	125ppm	Efficient	(15,16)
		Conductivity test	100ppm	Efficient	
Palm leave extract	CaCO_3_	Electrochemical measurements	75ppm	89.7	(16,17)
Bistorta Officinalis extract	Ca CO_3_	Electrochemical impedance spectroscopy	------	99.5	(18)
Brown sea algae extract	Ca CO_3_	Electrochemical measurements	100ppm	79.3	(20)
Clove extract	CaCO_3_	Electrochemical measurements	150ppm	68.7	(19)
		Conductivity test	100 ppm	80.4	
Sargassum latifolium extract	CaCO_3_	Electrochemical measurements	100 ppm	64.7	(33)

### Microbiological examination

#### Quantitative assessment by plate count agar test

To assess the microbiological quality and stability of aqueous rosemary extract over six months, a comprehensive microbiological evaluation was conducted, focusing on both enumeration and identification of microbial contaminants. The primary test employed was the Total Bacterial Count (TBC), a standard method for quantifying the overall microbial load in a product. Samples were cultured on plate count agar and incubated at 35 °C for 48 h, to determine the enumeration of Colony Forming Units (CFU/ml).

Initial testing at baseline (zero time) showed varying TBC levels depending on the presence of other compounds. The rosemary extract alone exhibited a TBC of 23.3 × 10^2^CFU/ml, when rhamnolipids were added, the count decreased to 19.2 × 10^2^CFU/ml suggesting a potential antimicrobial effect of this additive. Conversely, the combination of rosemary extract and chitosan further reduced TBC of 14 × 10^2^CFU/ml, demonstrating the enhanced antimicrobial efficacy of this combination.

Subsequent testing at the three-month mark showed a significant shift in the microbiological profile. Chitosan proved to be the most effective additive for controlling bacterial growth. The combination of rosemary extract and chitosan exhibited a Total Bacterial Count (TBC) of only 24.5 × 110^2^ FU/ml, significantly lower than the other treatments. In contrast, samples containing rosemary extract alone, or in combination with rhamnolipids, displayed substantially higher counts, exceeding 74.7 × 10³ and 65.5 × 1010^2^ FU/ml, respectively. These findings highlight the superior antimicrobial properties of chitosan within the extract matrix, demonstrating its greater efficacy in inhibiting bacterial proliferation compared to rhamnolipids.

The six-month assessment further emphasized on the impact of storage conditions on microbial stability. The rhamnolipids mixture recorded the highest TBC at 122.37 × 10^2^ CFU/ml, followed by the rosemary extract alone with 84.22 × 10^2^FU/ml. In contrast, the chitosan mixture, showed the lowest TBC within this group, recording 67.33 × 10^2^CFU/ml.

### Specific pathogenic bacteriological and fungal identification

To further evaluate the microbiological quality of the aqueous rosemary extract, beyond basic enumeration, a targeted Specific Pathogenic Bacteriological and Mycological Analysis was conducted. This analysis aimed to identify the presence of specific pathogenic microorganisms, differentiating them from potentially beneficial microbes. Such an approach is critical, as not all bacteria and fungi are harmful, some play constructive roles within a microbial ecosystem. The process involved culturing samples on various selective media designed to support the growth of specific pathogens, ensuring precise identification and characterization.

**Fig. 13:**
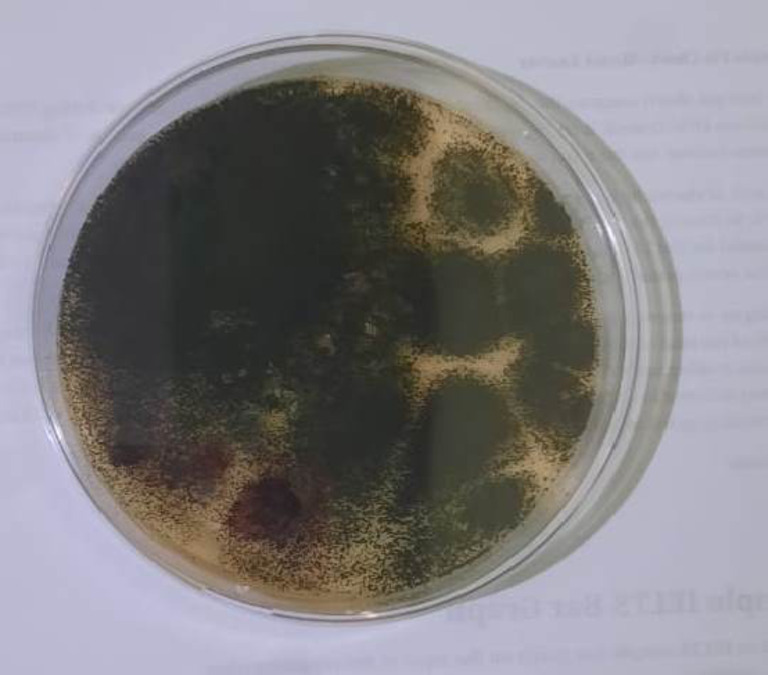
The *Aspergillus niger* black colonies on the Sabouraud Dextrose Agar (SDA) plates.

Initial Microbiological testing (complet the line with (zero time) showed no microbial growth on any media from all samples, confirming the baseline. After three months, the rosemary extracts alone remained free of any detectable pathogenic growth across all tested media (Table 5). However, the rosemary and rhamnolipids mixture exhibited black colonies on Sabouraud Dextrose Agar (SDA) (Fig. 13). These colonies were biochemically identified as *Aspergillus niger*, a common filamentous fungus. Notably, the rosemary-chitosan combination showed no microbial growth at this stage, reinforcing its antimicrobial potential.

**Table 5:** The bacteriological and mycological growth of the tested samples.

Type of used agar	Time	Type of tested inhibitors		
		Rosemary extract	Rosemary extract + Rhamnolipids	Rosemary extract + Chitosan
Mannitol Salt agar	3 months	-------------	-------------	-------------
	6 months	Pale colonies (*Staphylococcus epidermis*)	Pale colonies (*Staphylococcus epidermis*)	-------------
Sheep blood agar	3 months	-------------	-------------	-------------
	6 months	Pale non hemolytic colonies (*Staphylococcus epidermis*)	Pale non hemolytic colonies (*Staphylococcus epidermis*)	-------------
MacConkey agar	3 months	-------------	-------------	-------------
	6 months	-------------	-------------	-------------
Sabouraud Dextrose agar	3 months	-------------	Black colonies (*Aspergillus Niger*)	-------------
	6 months	Grayish colonies (*Aspergillus fumigatus*)	Grayish colonies (*Aspergillus fumigatus*)	-------------

The six-month assessment revealed notable changes in the mycological profile.

**Fig. 14:**
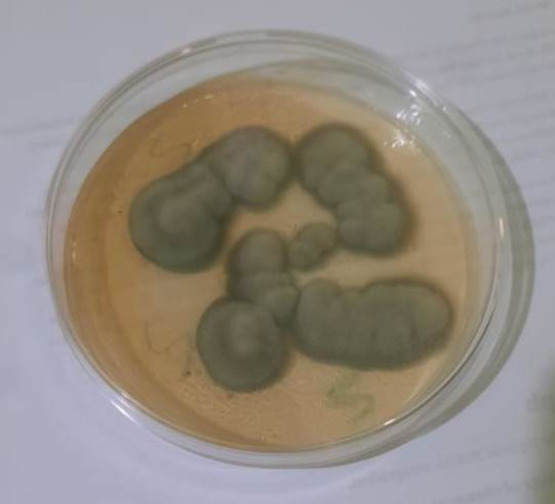
The *Aspergillus fumigatus* grayish colonies on the Sabouraud Dextrose Agar (SDA) plates.

On SDA, both the rosemary extract and the rosemary-rhamnolipids mixture produced grayish colonies. Microscopical and morphological analyses (Fig. 14) identified these colonies as *Aspergillus fumigatus*, a common opportunistic fungal pathogen.

**Fig. 15:**
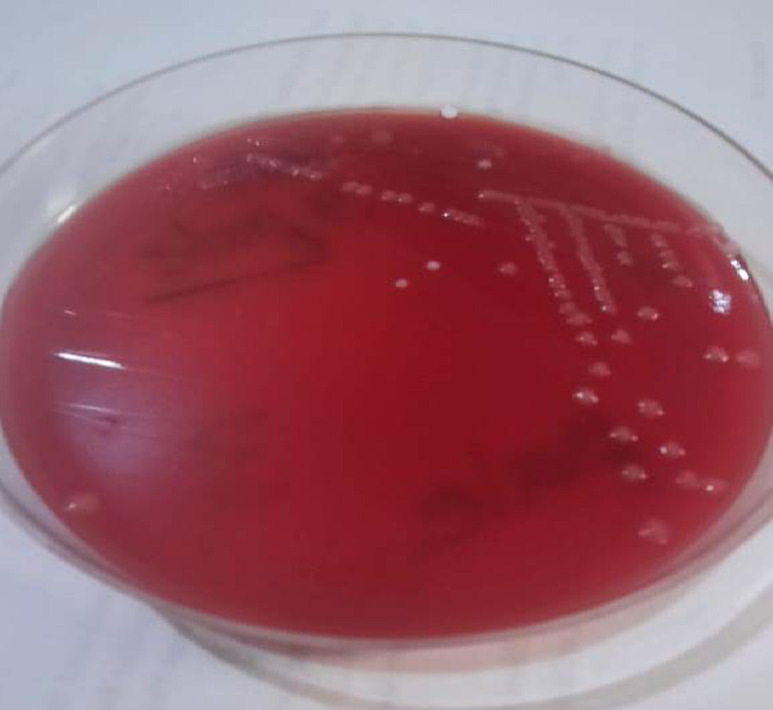
The *Staphylococcus epidermis* pale colonies on the Mannitol agar plate.

On Mannitol Salt Agar (MSA) and Sheep Blood Agar (SBA), pale colonies and pale non-hemolytic colonies, respectively, were observed in samples containing rosemary extract and the rosemary-rhamnolipids mixture. These colonies were biochemically identified as *Staphylococcus epidermidis* (Fig. 15), a Gram-positive, catalase-positive, and coagulase-negative bacterium. In contrast, the rosemary-chitosan mixture continued to exhibit no microbial growth of any tested medium, highlighting its potent antimicrobial activity.

This comprehensive approach, combining enumeration with identification, provides a robust understanding of the microbiological dynamics within the rosemary extract over time. The findings align with existing research demonstrating the antioxidant and antibacterial properties of rosemary extract, particularly its effectiveness against the Enterobacteriaceae family and Gram-negative bacteria^48^ The antimicrobial effects observed also align with the findings of Xing et al.^49^ and Yilmaz Atay^50^, who reported that chitosan inhibits *Escherichia coli* and *Staphylococcus aureus* by disrupting their cell membranes and targeting extracellular or intracellular sites. This mechanism likely explains the absence of microbial growth in the rosemary-chitosan mixtures throughout the six-month study period.

Safety Implications of Treatments:

Although total bacterial counts (TBC) increased in some treatments over the six months, the majority of bacteria identified were not pathogenic. For example, *Staphylococcus epidermidis*, an opportunistic pathogen commonly found in human epithelial microflora, was detected. Generally, *S. epidermidis* maintains a benign relationship with its host and may even have probiotic properties, as it can inhibit colonization by more pathogenic bacteria such as *Staphylococcus aureus*. However, evidence of *S. epidermidis* actively preventing colonization by other microorganisms in vivo remains inconclusive^51^.

Similarly, the pathogenic *Asperigellus* fungi isolated during the study, are naturally associated with plant-based products preserved over extended periods^52^. However, the rosemary-chitosan combination demonstrated superior results and reduced both pathogenic fungal and bacterial growth more effectively than other treatments. This makes it the most promising treatment option for applications requiring long-term microbial stability and safety.

## Materials and methods

### Preparation of solutions

Analytical reagent-grade NaCl, NaHCO_3_, Na_2_SO_4,_ and CaCl_2_ (Al-Gomhorya Chemicals CO., Egypt) were used for utilized to prepare solutions using double distilled water. A stock solution of rosemary extract was obtained by refluxing 5 g of ground sample of rosemary (*Salvia rosmarinus)* in 100 mL of distilled water at 100^o^C for one hour. After filtration of the refluxed solution, the concentration of the stock solution was determined in grams per liter, with a pH value of 5.91. The stock solution of rosemary extract was stored in sterilized clean glass bottles in the refrigerator at 4^o^ C.

### Electrochemical measurements for CaCO3 scaling

Calcium carbonate layer accumulation has been investigated electrochemically via chronoamperometry and electrochemical impedance spectroscopy techniques. This test used a three-electrode mode cell, with platinum wire (counter electrode) and a saturated calomel electrode (SCE) (reference electrode) for the electrochemical experiments in synthetic cooling water^53^. The working electrode steel’s chemical composition was as follows (wt%): Mn = 2.5, *P* = 0.04, Si = 0.35, C = 0.21, S = 0.04, and Fe in balance. To precipitate CaCO_3_ scales and measure electrochemical impedance, the Gamery instrument G300TM Potentiostat/Galvanostat/ZRA was utilized. To accelerate calcium carbonate precipitation, using the following equation, the steel electrode was polarized for three hours to − 0.95 V (vs. SCE) in 100 ml of the test solution, with a pH value of 7.37 and TDS, 47e^3^ mg/L.

3 $$Ca^{{2 + }} + {\text{ }}HCO_{3} ^{ - } + {\text{ }}OH^{ - } \textregistered CaCO_{3} \left( s \right){\text{ }} + {\text{ }}H_{2} O$$

Following the scale deposition process using the previous cathodic polarization, EIS measurements were carried out. EIS measurements were conducted between 0.1 and 1 × 104 Hz, with an applied potential signal amplitude of 10 mV. Every measurement was conducted at 40.0 ± 0.1 °C. In every instance, experiments were conducted in triplicate under identical conditions to assess the consistency and reliability of the experiments.

### Conductivity test for CaCO3 scaling

The conductivity test is a rapid test used in this work to monitor the anti-scaling performance of the suggested materials over time. In line with previous research, a conductivity test was conducted^54^. A solution of 5 mL 0.1 M CaCl_2_ was mixed with a specific volume of the tested inhibitor, and the mixture was diluted to 100 mL with distilled water with a TDS value of 540–612 mg/L according to the inhibitor. The pH values ranged from 6.00 to 6.4 except in the case of chitosan addition, the pH value dropped to 4.4. The conductivity of the stirred solution was monitored during titration with 0.1 M Na_2_CO_3_ using a HANNA conductivity meter. The titrant was added in 0.2 mL increments. The measurements were performed at 25.0 ± 0.1 °C. Experiments were conducted in triplicate under identical conditions to assess the consistency and reliability of the experiments.

### Morphological examination of precipitated CaCO3 crystals

The CaCO_3_ scale morphology and crystal lattices were examined using a JEOL-5300 scanning electron microscope (SEM). The scale crystals underwent a vacuum sputter coating process to apply a thin layer of gold before being analyzed with the SEM.

The crystallinity of the CaCO_3_ scale was characterized using X-ray powder diffractometer-XRD-D2 phaser, Bruker, Germany, using Cu Ka radiation (40 kV and 30 mA) and an Ni filter with a scanning speed of 0.005° 2q s21. The time constant was set at 2 s.

### FTIR analysis

Using an FT-IR spectrometer (Perkin-Elmer LS-55-Luminescence Spectrometer), the produced extract’s chemical composition was examined. After the solutions were dried, the KBr pellet technique was used to characterize the dried powders in the region of 4000–500 cm^− 1^.

### Microbiological examination

#### Quantitative assessment using plate count agar method

The bacteriological enumeration test was performed following the standard procedure for the plate count agar method. Four treatment groups were prepared, each with three replicated samples:

1. Blank control, contained no extract.
2. Rosemary extract only, including aqueous rosemary extract.
3. Rosemary with Rhamnolipids mixture, combined rosemary extract with rhamnolipids (2:1).
4. Rosemary with Chitosan mixture, combined rosemary extract with chitosan (2:1).

All samples were incubated at 35ºC for 48 h^55^ to determine the bacterial load by enumerating Colony Forming Units (CFU/ml).

### Specific pathogenic bacteriological and fungal analysis

About 1 ml of each sample was incubated for 24 h at 37 °C in a suspension in 25 ml of peptone water^56^. Bacteria were isolated by streaking the pre-enriched culture from the peptone water onto three different types of agar media. Using Mannitol Salt Agar (MSA), the *Staphylococcus* species were identified. MacConkey agar (MA) was additionally used to check for the presence of bacteria from the *Enterobacteriaceae* family, including *Salmonella* and *E. coli* species. The samples were also examined for the presence of other pathogenic bacteria, such as *Streptococcus* species, using Sheep Blood Agar (SBA). At 37 °C, all of the plates underwent a 24- to 48-hour aerobic incubation period.

Following the procedures outlined by Oyeleke and Manga^57^. The pure isolates underwent additional characterization and identification based on cell morphology, Gram stain, and biochemical assays. The Gram stain was applied to the colonies that were chosen from NA. The catalase test was used to distinguish between the gram-positive cocci species *Staphylococcus* spp. and *Streptococcus* spp. On colonies that tested positive for catalase, slide, and tube coagulase tests were also carried out to establish the presence of *Staphylococcus aureus*^58^.

By streaking on Sabouraud Dextrose Agar (SDA) plates, which were incubated for 4–7 days at 37 °C, fungal contamination was identified. The isolates were then identified using Domsch and Gams’s methods^59^.

## Conclusions

This research represents a novel approach for extending the shelf life of naturally based water treatment products. The findings indicate that *Salvia rosmarinus* extraction can effectively inhibit calcium carbonate scaling. The effectiveness of rosemary extract may be attributed to various functional groups, such as carboxylate and amides, which serve as adsorption sites on crystal embryos distorting their structure and preventing their growth. Chitosan and rhamnolipids were evaluated as two bio-preservatives, representing polysaccharides and biosurfactants, respectively. The combination of rosemary extracts and chitosan in a 2:1 ratio proved to be a highly effective green scale inhibitor, outperforming rhamnolipids. Rosemary extract-chitosan mixture inhibited calcium carbonate scale formation with efficiencies of 82% at initial measurement and 80% after six months. Microbiological examination revealed that combining the rosemary extract with chitosan produced the best results and reduced pathogenic bacteria or fungi. Overall, the beneficial properties of biosurfactants and polysaccharides position them as viable biological alternatives to toxic and chemical biocides in naturally based water treatment production.

## Electronic supplementary material

Below is the link to the electronic supplementary material.

Supplementary Material 1

## Data Availability

Data is provided within the manuscript or supplementary information files.
