# Supplementary material for: Enhancing the shelf life of natural scale inhibitors using bio preservatives
Source: Sci Rep. 2025 Mar 8;15:8115. doi: 10.1038/s41598-025-90831-5 (PMC11890854; doi:10.1038/s41598-025-90831-5)
Supplement: Supplementary file 1 — Supplementary Material 1 [file 41598_2025_90831_MOESM1_ESM.docx]

**Supplementary data**


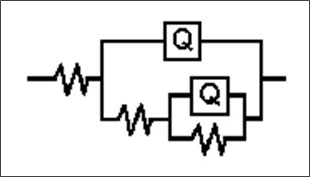


**Fig.S1:** Schematic for the equivalent circuit model used.

**Fig.S2**: Variation of Conductivity of 0.1M CaCl_2_ solution in the absence and presence of different concentrations of Rosemary extract at 25°C.

**Table S1**: Efficiency of rosemary extract, rosemary extract with rhamnolipids & rosemary with chitosan in ratio(20:1) through 6 months as CaCO_3_ inhibitor

| **24 weeks** | **20 weeks** | **16 weeks** | **12 weeks** | **10 weeks** | **8 weeks** | **6 weeks** | **4 weeks** | **3 weeks** | **2 weeks** | **One week** | **Zero time** |  |
| --- | --- | --- | --- | --- | --- | --- | --- | --- | --- | --- | --- | --- |
| 67.8 | 67.8 | 68.8 | 68.8 | 70 | 70 | 70 | 73 | 75.5 | 75.5 | 75.5 | 78.3 | Rosemary extract |
| 63 | 63 | 65 | 68.9 | 68.9 | 68.9 | 70 | 70 | 75.5 | 75.5 | 75.5 | 78.3 | Rosemary extract +Rhamnolipids |
| 74.2 | 74.2 | 74.2 | 76.3 | 77.5 | 77.5 | 79.5 | 79.5 | 80 | 80 | 80 | 80 | Rosemary extract +Chitosan |

**Table S2**:Efficiency of rosemary extract ,rosemary extract with rhamnolipids & rosmary extract with chitosan in ratio(2:1) through 6 month as CaCO_3_ inhibitor

| **24 weeks** | **20 weeks** | **16 weeks** | **12 weeks** | **10 weeks** | **8 weeks** | **6 weeks** | **4 weeks** | **3 weeks** | **2 weeks** | **One week** | **Zero time** |  |
| --- | --- | --- | --- | --- | --- | --- | --- | --- | --- | --- | --- | --- |
| 67.8 | 67.8 | 68.8 | 68.8 | 70 | 70 | 70 | 73 | 75.5 | 75.5 | 75.5 | 78.3 | Rosemary extract |
| 70.9 | 70.9 | 70.9 | 71.8 | 71.8 | 71.8 | 72.72 | 72.72 | 75.5 | 75.5 | 75.5 | 78.3 | Rosemary extract +Rhamnolipids |
| 80 | 80 | 80 | 80 | 80 | 80 | 80 | 81.6 | 81.6 | 82 | 82 | 82 | Rosemary extract +Chitosan |
